# Deep learning identifies a T-cell exhaustion-dependent transcriptional signature for predicting clinical outcomes and response to immune checkpoint blockade

**DOI:** 10.1038/s41389-023-00482-2

**Published:** 2023-07-11

**Authors:** Zicheng Zhang, Hongyan Chen, Dongxue Yan, Lu Chen, Jie Sun, Meng Zhou

**Affiliations:** School of Biomedical Engineering, Eye Hospital, Wenzhou Medical University, 325027 Wenzhou, China

**Keywords:** Tumour biomarkers, Cancer microenvironment

## Abstract

Immune checkpoint blockade (ICB) therapies have brought unprecedented advances in cancer treatment, but responses are limited to a fraction of patients. Therefore, sustained and substantial efforts are required to advance clinical and translational investigation on managing patients receiving ICB. In this study, we investigated the dynamic changes in molecular profiles of T-cell exhaustion (TEX) during ICB treatment using single-cell and bulk transcriptome analysis, and demonstrated distinct exhaustion molecular profiles associated with ICB response. By applying an ensemble deep-learning computational framework, we identified an ICB-associated transcriptional signature consisting of 16 TEX-related genes, termed ITGs. Incorporating 16 ITGs into a machine-learning model called MLTIP achieved reliable predictive power for clinical ICB response with an average AUC of 0.778, and overall survival (pooled HR = 0.093, 95% CI, 0.031–0.28, *P* < 0.001) across multiple ICB-treated cohorts. Furthermore, the MLTIP consistently demonstrated superior predictive performance compared to other well-established markers and signatures, with an average increase in AUC of 21.5%. In summary, our results highlight the potential of this TEX-dependent transcriptional signature as a tool for precise patient stratification and personalized immunotherapy, with clinical translation in precision medicine.

## Introduction

Immune checkpoint blockade (ICB) therapy has revolutionized cancer therapy by disrupting co-inhibitory T-cell signaling unprecedentedly, leading to a stupendous response and improved survival for certain patients with various cancers [1]. However, monoclonal antibodies for immune checkpoints, such as targeting programmed cell death protein 1 (PD-1), programmed death-ligand 1 (PD-L1) and cytotoxic T-lymphocyte-associated protein 4 (CTLA-4), have displayed heterogeneous and variable response rates and clinical outcomes in a large proportion of cancers [2–4]. Therefore, it is critical to identify predictive biomarkers of individual ICB responses and therapeutic targets to improve tailored clinical decisions and treatment procedures. Although potential biomarkers of ICB response have been reported and investigated over the past years, such as immune checkpoint inhibitor gene expression [5], IFN-γ pathway [6], tumor-infiltrating lymphocytes [7], tumor mutation burden [8], T-cell receptor [9], CTLA-4 promoter hypomethylation [10], DNA repair machinery [11], microsatellite instability [12], neoantigen presentation [13], sex differences [14, 15], the gut microbiome [16], and immune-related adverse events [17], these previously markers are insufficient and limited by moderate accuracy, cancer heterogeneity and tissue specificity. Therefore, sustained and substantial efforts are yet required to advance clinical and translational investigation on managing patients receiving ICB.

There is mounting evidence to suggest that the reversion of the T-cell exhaustion (TEX) state and progressive characteristics from "stem cell-like progenitor" to "terminally differentiated exhausted cell" are critical factors involved in response resistance to ICB therapy [18, 19]. In the tumor microenvironment (TME), the complex networks of immunosuppressive and escaping cancer cells, inflammatory cells, suppressive cytokines, and chemotactic factors induce differentiation from effector T cells into exhausted T cells [20]. The intrinsic mechanism of evolution for T-cell exhaustion within TME can be attributed to multiple factors, including evolutionary conservative adaptation of chronic tumor antigen stimulation [21], chronic T-cell receptor (TCR) stimulation [22], and epigenetic and transcriptional reprogramming [23, 24]. Although increased expression of various inhibitory receptors is the hallmark of exhausted CD8 + T cells, the pool of exhausted CD8 + T cells is composed of functionally and phenotypically heterogeneous subsets [25, 26]. The heterogeneity of TEX within the TME has been reported to be associated with clinical outcomes and immunotherapy responsiveness [25]. For example, a recently discovered precursor exhausted T-cell subset, a specialized population of exhausted CD8 + T cells with dual characteristics of both exhausted and memory cells, is closely linked to increased ICB response and patient survival [27, 28]. Another TEX subpopulation marked by CD62L expression (CD62L + TEX cells) also plays a critical role in long-term antiviral immunity and responsiveness to immunotherapy [25].

In this study, we focused on the close relationship between transcriptional alterations in TEX and ICB response. Using deep-learning algorithms on transcriptome data and ICB response data, we aimed to identify a TEX-dependent transcriptional signature that could predict clinical outcomes and response to ICB. Our analysis resulted in the discovery of a TEX-dependent transcriptional signature, which we named MLTIP. We found that the MLTIP could effectively predict clinical outcomes and ICB responses. We then validated the predictive capacity of the MLTIP using a comprehensive dataset of 465 samples sourced from five multi-center ICB immunotherapy cohorts. Furthermore, we found that the MLTIP had superior performance and outperformed other established signatures.

## Materials and methods

### Acquisition and analysis of ICB bulk RNA-seq datasets

The raw bulk RNA-seq data was obtained using the HiSeq Illumina platform from six ICB cohorts, which were collected from the NCBI Sequence Read Archive (SRA) repository and other online sources, including 41 patients treated with anti-PD-1 from Gide’s study (Accession PRJEB23709) (Gide cohort) [29], 49 patients treated with anti-PD-1 and 42 paired pre- and post- anti-PD-1 treatment patients from Riaz’s study (Accession PRJNA356761) (Riaz cohort) [30], 26 patients treated with anti-PD-1 from HugoW’s study (HugoW cohort) (Accession PRJNA312948) [31], nine patients with anti-CTLA-4 immunotherapy from Nathanson’s study (http://www.hammerlab.org/melanoma-reanalysis) (Nathanson cohort) [32], and 298 patients with anti-PD-L1 immunotherapy from Maria’s study (http://research-pub.gene.com/IMvigor210CoreBiologies) (Maria cohort) [33]. After batch correction using the Combat method from “sva” R package, Gide cohort, Riaz cohort, and HugoW cohort were integrated to form a larger cohort (referred to as GHR cohort) for discovery, and other cohorts were used for validation. Clinical benefit was defined as patients with a RECIST complete response (CR), and stable disease (SD), while non-responders were defined as those with progressive disease (PD).

Raw bulk RNA-seq data of ICB cohorts was converted into FASTQ data from SRA using “sratoolkit” (v2.8.2). Quality control was performed on the FASTQ files using “Trim Galore”. Gene expression quantification was performed using “kallisto”. Raw count data was transformed by each gene length in kilobases of transcripts per million (TPM). The TPM expression was normalized using the log2(TPM) quantification approach suggested by the center of the University of North Carolina TCGA genome characterization. After gene expression normalized by log2x, RNA-seq data were subjected to R packages “tsne” (v0.1–3), “stats” (v4.1.1) and “ConsensusClusterPlus” (v1.56.0) using the Euclidean distance, ward.D, and k-means method of TEX genes.

### Pan-cancer transcriptomic data

The TCGA pan-cancer RNA-seq FPKM-UQ (log_2_ FPKM + 1 transformed) along with curated clinical information for 9564 patients among 30 cancer types (adrenocortical carcinoma (ACC), bladder urothelial carcinoma (BLCA), breast invasive carcinoma (BRCA), cervical squamous cell carcinoma and endocervical adenocarcinoma (CESC), cholangiocarcinoma (CHOL), colon adenocarcinoma (COAD), esophageal carcinoma (ESCA), glioblastoma multiforme (GBM), head and neck squamous cell carcinoma (HNSC), kidney chromophobe (KICH), kidney renal clear cell carcinoma (KIRC), kidney renal papillary cell carcinoma (KIRP), brain lower grade glioma (LGG), liver hepatocellular carcinoma (LIHC), lung adenocarcinoma (LUAD), lung squamous cell carcinoma (LUSC), mesothelioma (MESO), ovarian serous cystadenocarcinoma (OV), pancreatic adenocarcinoma (PAAD), prostate adenocarcinoma (PRAD), rectum adenocarcinoma (READ), sarcoma (SARC), skin cutaneous melanoma (SKCM), stomach adenocarcinoma (STAD), testicular germ cell tumors (TGCT), thyroid carcinoma (THCA), thymoma (THYM), uterine corpus endometrial carcinoma (UCEC), uterine carcinosarcoma (UCS), uveal melanoma (UVM) was obtained from the UCSC Xena atlas (https://xenabrowser.net/datapages/ (GDC Pan-cancer)). The 9564 samples were only filtered with primary and metastatic bulk solid tumors.

### Single-cell RNA-seq data and analysis

10x Genomics’ single-cell RNA-seq (scRNA-seq) data of triple-negative breast cancer (TNBC) receiving anti-PD-L1 immunotherapy was obtained from the Zhang cohort [34], including 52,960 T cells from 14 pre-treatment patients and 43,401 T cells from 12 post-treatment patients. The quantified normalized T-cell scRNA-seq subtype data from the Zhang cohort was processed using the “Scanpy” (v2.3.4) [35] and “Scrublet” [36] methods to reserve the high-quality cells. The R package “Seurat” (v4.1.0) [37] was utilized to perform the reasonable clustering of the 52,960 T cells in pre-treatment and 43,401 T cells in post-treatment with their top highly variable genes (HVGs). The HVGs were detected with the “selection.method=vst” in the Seurat package, where the normalized dispersion using the observed mean and expected variance revealed the gene dispersions. For downstream analysis, the principal component analysis (PCA) was carried out for dimensionality reduction and noise reduction. The cell clustering used non-linear dimension reduction to the same principal components with the t-Distributed Stochastic Neighbor Embedding (t-SNE) in the two-dimensional space [37]. The “FindAllMarkers” function in the Seurat package was performed to identify the differentially expressed genes (DEGs) between each cell cluster and all other cell clusters with the threshold of 0.25 logFC and *P* value < 0.05. The overlap between these DEGs and candidate cellular biomarkers was displayed in the heatmap with the scaled average gene expression.

### TEX-dependent predictor with the deep-learning ensemble structure

A total of 683 genes involved in TEX were obtained from previous studies by manual collection (Supplementary Table 1). A deep-learning ensemble structure was established to construct a TEX-based predictor by integrating deep-autoencoder [38], Kruskal–Wallis test method, and recursive feature elimination (RFE) as follows: (i) The deep-autoencoder was used for the noise reduction and feature-selection with “hidden=41” by the encoder to decoder. For each node in the layer of the deep-autoencoder neural network, the “Tanh” was used as the activation function, and the “Automatic” was performed as the loss function from the encoder to the decoder. The importance threshold of the candidate feature was 10%. (ii) The built-in Kruskal–Wallis test in R package “stats” (v4.1.1) was utilized to identify DEGs among four RECIST states (CR, PR, SD, and PD) of patients. The significant differential threshold was *P* < 0.05. (iii) The machine-learning model was built using the built-in RFE in R package “caret” (v6.0-90, https://github.com/topepo/caret/) to filter out the poor feature in each iteration. External resampling analysis of RFE was conducted using the threefold cross-validation. The candidate features were determined by the threshold of overall importance >0 among four RECIST states.

Finally, a crucial informative gene list was designed for calculating the TEX predictor score using the machine-learning model eXtreme Gradient Boosting (XGBoost, R package v1.5.2.1) with the kernel function of “binary-logistic"'. The evaluation metrics of XGBoost were built using “error” (error for classification), and the threshold of “subsample” and “colsample_bytree” were both 0.5 to avoid overfitting. The maximum number of boosting iterations was ten and the maximum tree depth was 5. The alpha L1 and L2 regularization terms were 0 and 1 on weight, respectively.

### Pathway analysis and functional annotation

The single-sample Gene Set Enrichment Analysis (ssGSEA) of R package “GSVA” (v1.40.1) [39] was applied to the log2-transformed normalized TPM expression to infer the absolute enrichment score for four major TEX-related pathways [interferon-gamma (IFN-γ), tumor necrosis factor (TNF), interleukin-2 (IL-2), cytotoxic T lymphocytes (CTL)], 24 immune response pathways and 28 immune cell types from Molecular Signatures Database (MSigDB, v7.2) [40], and previous Pornpimol’s genotype-immunophenotype study [41] on multi-center cohorts and TCGA pan-cancer cohort. The functional annotation of the KEGG pathway, GO-biological process (BP), GO-molecular function (MF) and GO-cellular component (CC) were performed by “clusterProfiler” (v4.0.5) [42] with genome-wide annotation for Human “org.Hs.eg.db” (v3.13.0, https://www.bioconductor.org/packages/release/data/annotation/html/org.Hs.eg.db.html).

### Survival analysis

For the clinical information of patients, including overall survival time (OS) and progression-free survival time (PFS), the univariable Cox analysis was performed to identify the prognostic risk factor, which emerged with a hazard ratio (HR), 95% confidence interval (95% CI) and P-values. The Kaplan–Meier survival curves were used to reveal the difference among subgroups using the log-rank test, and the significance was presented with *P* value. All methods were implemented using the R package “survival” (v3.3-0) and the *P* value < 0.05 was delimited as statistically significant.

### Statistical analysis

Apart from the bioinformatics mentioned above methods, all statistical analyses in this study were conducted using R (v4.1.1) on the RStudio platform (v1.4.1717), along with associated packages. Except some of Fisher’s exact tests were one-sided alternatives, other statistical tests were two-sided. Differences among subgroups were examined using Fisher’s exact tests, chi-squared tests, Wilcoxon tests, paired-samples *t* tests, and Kruskal–Wallis tests. The performance of the predictor as a classifier for ICB immunotherapy response was assessed with receiver operating characteristic curves (ROC) with the area under the ROC curve (AUC), 95% confidence interval (95% CI), specificity and sensitivity in “pROC” (v1.18.0) R package. When a *P* value reported by R (v4.1.1) was smaller than 0.001, it was portrayed as “*P* < 0.001”.

## Results

### Association of transcriptional changes in TEX with ICB response

**Fig. 1:**
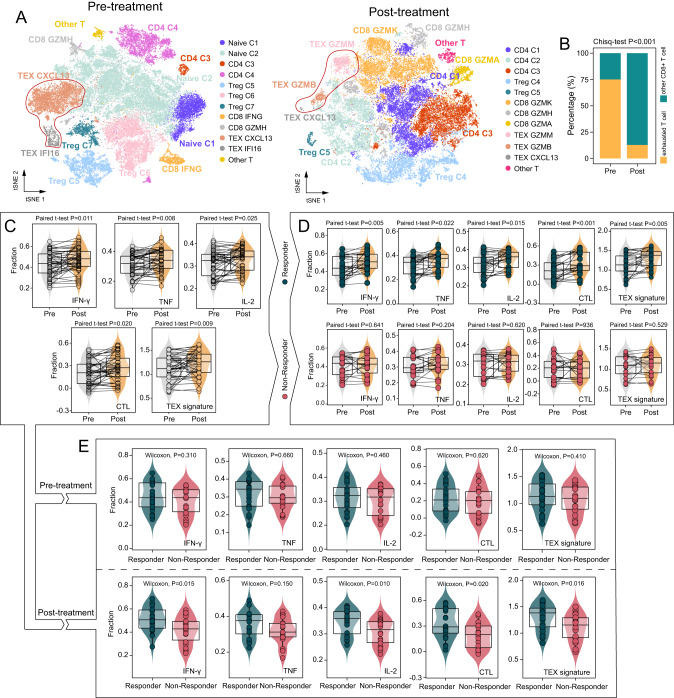
Distinct exhaustion profiles of CD8 + T cell during ICB treatment. **A** T-distributed stochastic neighbor embedding (t-SNE) visualization of T-cell clusters (52,960 cells for 14 pre-treated patients and 43,401 cells for 12 post-treated patients) with specific markers, showing the annotation and color nodes for T-cell subtypes in the tumor ecosystem. **B** The proportion of exhausted T cells and other CD8 + T cells between pre-treated and post-treated BC patients (Chi-square test). **C** Box plots showing computationally estimated activities of TEX signature and pathways in paired ICB-treated melanoma patients (Two-sided paired *t* test). **D** Box plots showing changes in the computationally estimated activity of TEX signature and pathways before and after ICB treatment for responders and non-responders, respectively (two-sided paired *t* test). **E** Box plots showing the difference in computationally estimated activities of TEX signature and pathways between responders and non-responders before and after ICB treatment (Wilcoxon test).

To delineate the transcriptional changes of TEX during ICB therapy at single-cell resolution, we analyzed single-cell transcriptomic profiles of 52,960 and 43,401 high-quality T cells between pre- and post-ICB TNBC patients from the Zhang cohort [34], and grouped T cells into 12 subsets using the t-SNE (Fig. 1A and Supplementary Fig. 1). After ICB treatment, a significant difference was observed in the relative abundance of exhausted T cells versus other CD8 + T cells (Chi-square test *P* < 0.001). Specifically, we observed 6546 exhausted T cells (TEX CXCL13 and TEX IFI16) and 2177 CD8 + T cells in pre-ICB patients, and 1733 exhausted T cells (TEX CXCL13, TEX GZMM, and TEX GZMB) and 11,802 CD8 + T cells in post-ICB patients (Fig. 1B). Upon analyzing bulk-tissue transcriptomic profiles of paired ICB-treated melanoma patients from the Riaz cohort, we detected a significant increase in enrichment scores of TEX-related signatures and pathways after ICB treatment (Fig. 1C). However, further investigation of transcriptional changes of TEX with ICB response illustrated a significant increase in enrichment scores of TEX-related signatures and pathways solely in responders, but not in non-responders after ICB treatment (Fig. 1D). Remarkably, there were no significant differences in the enrichment of TEX-related signature and pathways before ICB treatment between responders and non-responders. However, after ICB treatment, responders exhibited higher enrichment scores of TEX-related signature and pathways compared to non-responders (Fig. 1E). These findings substantiate the link between transcriptional changes of TEX and response to ICB immunotherapy.

### Deep-learning identification of a TEX-dependent transcriptional signature associated with ICB response

**Fig. 2:**
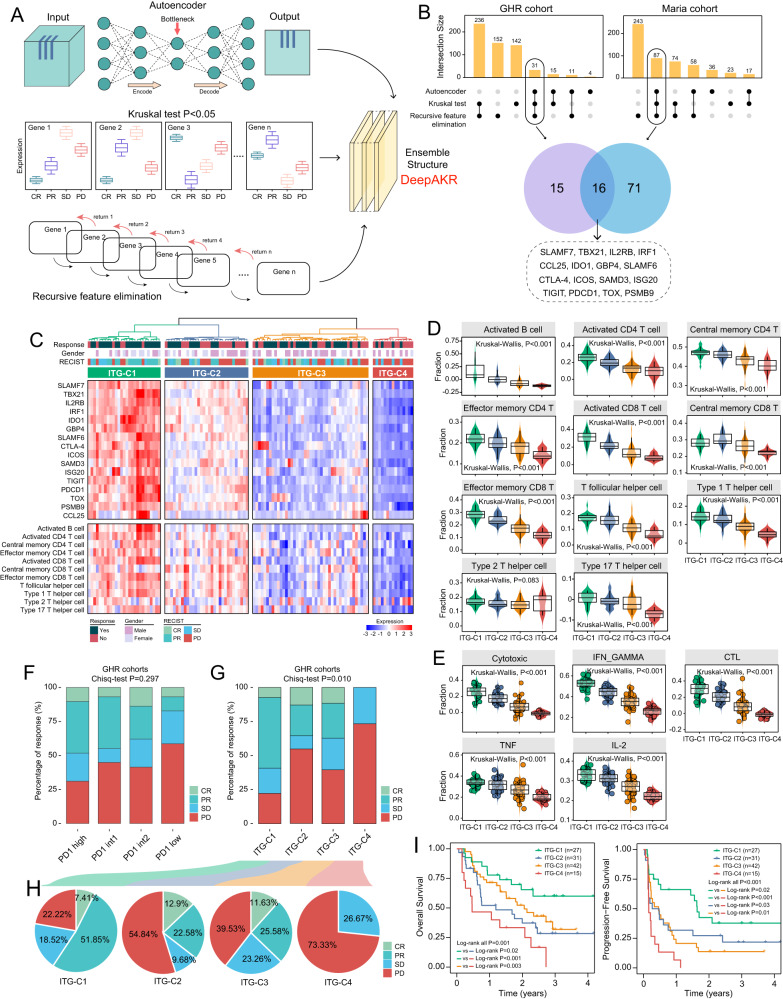
Deep-learning identification of transcriptional program associated with TEX heterogeneity and ICB response. **A** Workflow of an ensemble deep-learning computational framework. **B** Bar plots showing the transcriptional programs with deep learning in the GHR and Maria cohorts. Venn diagram showing the overleaping transcriptional programs between the GHR and Maria cohorts. **C** Unsupervised hierarchical clustering heatmap of 116 melanoma tumors using expression pattern of 16 ICB response-associated TEX genes (ITGs). **D** Box plots showing the monotonic association between the computationally estimated abundance of tumor-infiltrating immune cells and ITG subtypes (Kruskal–Wallis test). **E** Box plots showing the monotonic association between the computationally estimated activity of immune-related biometrics and ITG subtypes (Kruskal–Wallis test). **F**, **G** Histograms showing the percentage of each RECIST archetype (CR/PR/SD/PD) among four PD-1 subgroups (**F**) and ITG subtypes (**G**) (Chi-squared test). **H** Pie charts showing the distribution of each RECIST (CR/PR/SD/PD) archetype in four ITG subtypes from the GHR cohort. **I** Kaplan–Meier curves comparing OS and PFS among four ITG subtypes (log-rank test).

We developed an ensemble deep-learning computational framework, DeepAKR, to identify transcriptional program underlying TEX and ICB response through imbedding the transfer-learning design with supervised pre-training using the RESTIC labeled tumor samples to learn TEX characterizations in melanoma cohort (GHR cohort), followed by parameters fine-tuning on metastatic urothelial carcinoma (mUC) cohort (Maria cohort) to capture the association between ICB response and TEX-related genes (Fig. 2A). Finally, the DeepAKR identified 16 ICB response-associated TEX genes, referred to as ITGs, including *SLAMF7*, *TBX21*, *IL2RB*, *IRF1*, *CCL25*, *IDO1*, *GBP4*, *SLAMF6*, *CTLA-4*, *ICOS*, *SAMD3*, *ISG20*, *TIGIT*, *PDCD1*, *TOX*, and *PSMB9* (Fig. 2B). Based on the expression pattern of these genes, unsupervised hierarchical clustering classified 116 melanoma tumors into four clusters (ITG-C1 to ITG-C4) with decreasing expression trend of 16 ITGs from ITG-C1 to ITG-C4 (Fig. 2C). We observed significant differences in T and B lymphocytes composition of the TIME between the four clusters (Kruskal–Wallis test, *P* < 0.001) (Fig. 2D). Tumors of ITG-C1 and ITG-C2 had high infiltration abundance of lymphocytes, whereas ITG-C3 and ITG-C4 had low infiltration abundance. Furthermore, we scored five TEX-related gene signatures (Cytotoxic, IFN-γ, TNF, IL-2, and CTL) using ssGSEA and found decreasing tendency from ITG-C1 to ITG-C4 (Kruskal–Wallis test, *P* < 0.001) (Fig. 2E).

The GHR melanoma solid tumors displayed four major phenotypes using equipartition based on the expression levels of PD-1, and the remaining new subgroups were assigned to these four categories (Fig. 2F). The quantitative analysis of RECIST scores for melanomas revealed that the PD-1 high subgroup had the lowest proportion of progressive disease (PD) state (31.03%), while the PD-1 low cluster exhibited the highest rate of PD state (58.62%). However, there were no significant differences in RECIST scores among the four subtypes of PD-1 expression (Fig. 2F, Chi-squared test *P* = 0.297). As for the association of the response percentage with ITG clusters, we observed a significant difference in RECIST distribution among four ITG clusters (Fig. 2G, Chi-squared test *P* = 0.010). The lowest proportion of PD states was found in the ITG-C1 cluster (22.22%), even lower than that in the PD-1 high subtype (31.03%). Meanwhile, the highest proportion of PD states was observed in the ITG-C4 subgroup (73.33%), which was higher than that in the PD-1 low subgroup (58.62%) (Fig. 2H). Moreover, survival analyses revealed that tumors from different ITG clusters in the GHR cohort exhibited significantly different overall survival (OS) and progression-free survival (PFS) (log-rank *P* < 0.001), with ITG-C1 showing improved considerably survival and ITG-C4 indicating the poorest survival (Fig. 2I).

### TEX-dependent machine-learning predictor of response to ICB immunotherapy

**Fig. 3:**
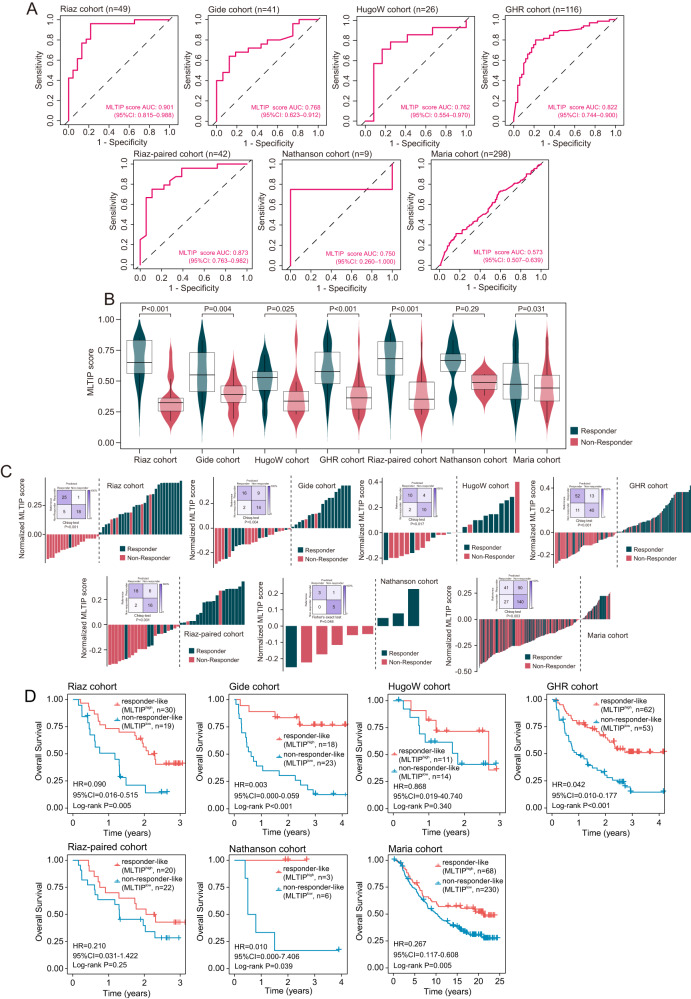
Performance of TEX-derived machine-learning predictor of response to ICB immunotherapy. **A** ROC curves and corresponding AUC values of MLTIP score. **B** Box plots showing the distribution of MLTIP scores between responders and non-responders (Wilcoxon test). **C** Waterfall plot of MLTIP scores and confusion matrices indicating predicted outcomes generated by MLTIP (Chi-square test). **D** Kaplan–Meier curves comparing OS between responder-like or non-responder-like groups predicted by the MLTIP.

Given the observed association of ITGs with ICB response, we developed a TEX-dependent predictor (MLTIP) of response to ICB immunotherapy by utilizing the XGBoost machine-learning method to integrate the 16 ITGs. We trained the MLTIP using the Riaz cohort and then validated the MLTIP in six independent ICB-treated cohorts. We calculated the MLTIP score for each sample and performed a ROC curves analysis using the MLTIP score to assess the predictive power. As shown in Fig. 3A, the MLTIP exhibited superior performance in predicting response to ICB immunotherapy across different cohorts, with AUCs of 0.901, 0.768, 0.762, 0.822, 0.873, 0.750, and 0.573 for the cohorts of Riaz, Gide, HugoW, GHR, Riaz-paired, Nathanson and Maria, respectively. Moreover, the MLTIP scores significantly differed between responders and non-responders, with responders showing higher scores than non-responders. (Fig. 3B). When dichotomizing MLTIP score predictions into either predicted responder-like or predicted non-responder-like groups, the MLTIP showed superior discriminatory power in distinguishing responders from non-responders, with the accuracy of 87.8%, 73.2%, 76.9%, 79.3%, 81%, 88.9%, and 60.7% for the cohorts of Riaz, Gide, HugoW, GHR, Riaz-paired, Nathanson, and Maria, respectively (Fig. 3C). Furthermore, significant differences in OS between predicted responder-like or non-responder-like groups. Tumors in the responder-like group predicted by the MLTIP had significantly better OS compared to tumors classified as non-responders with HR of 0.090, 0.003, 0.868, 0.042, 0.210, 0.010, and 0.267 for the cohorts of Riaz, Gide, HugoW, GHR, Riaz-paired, Nathanson, and Maria, respectively (Fig. 3D).

### Comparisons to other well-established markers and signatures

**Fig. 4:**
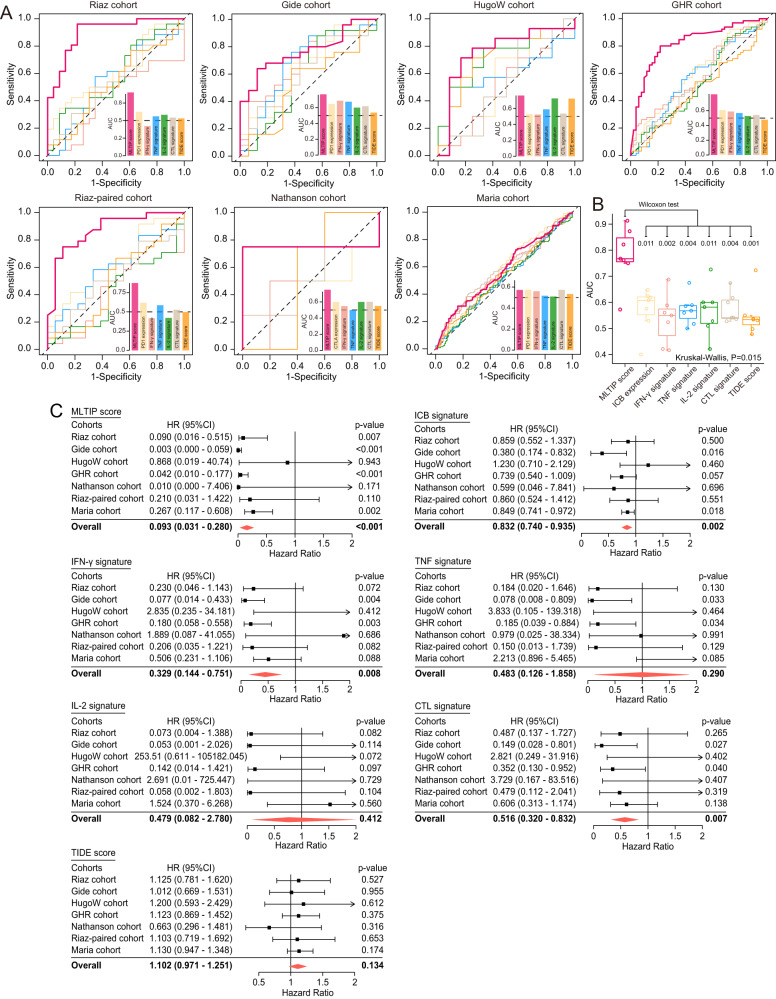
Performance comparisons to other well-established markers and signatures. **A** ROC curves, corresponding AUC values of MLTIP score, and other well-established markers and signatures. **B** Box plots showing the difference of AUC value between MLTIP scores and other well-established markers or signatures. **C** Forest plot visualizing HRs of univariate Cox regression analysis of OS in seven cohorts. The red diamond shows the meta-analysis summary of HRs over seven cohorts.

We compared the predictive performance of the MLTIP with immune checkpoints (PD-1, PD-L1, and CTLA-4), TMB burden, and six recently proposed signatures in predicting ICB response. The results of ROC analysis demonstrated that the MLTIP consistently achieved superior predictive performance (average AUC = 0.778) compared to other well-established markers and signatures across different cohorts (Fig. 4A, B and Supplementary Fig. S2).

We further compared the prognostic value of the MLTIP with that of six recently proposed signatures in multiple patient cohorts. We first used the univariate Cox regression analysis to evaluate the association of these signatures and the MLTIP with OS for each cohort. Then, we used a meta-analysis to leverage the multiple cohorts for an overall prognostic evaluation of each signature. As shown in Fig. 4C, although the MLTIP and other three signatures (ICB genes, IFN-γ signature, and CTL signature) showed a significant correlation with OS, the MLTIP (HR = 0.093, 95% CI, 0.031–0.280, *P* < 0.001) demonstrated better predictive performance in OS when compared with ICB genes (HR = 0.832, 95% CI, 0.740–0.935, *P* = 0.002), IFN-γ signature (HR = 0.329, 95% CI, 0.144-0.751, *P* = 0.008) and CTL signature (HR = 0.516, 95% CI, 0.320–0.832, *P* = 0.007).

### Association of the MLTIP with immune milieu and prognosis in pan-cancer

**Fig. 5:**
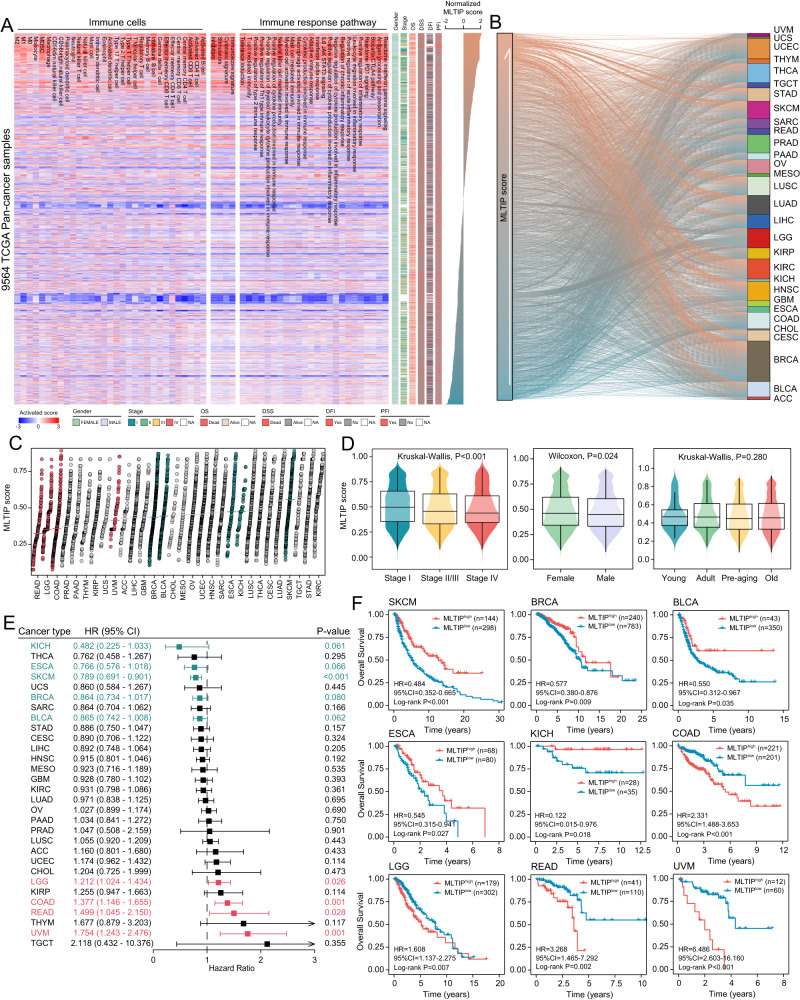
Pan-cancer characterization of the MLTIP with immune milieu and prognosis. **A** Heatmap of computationally estimated fraction for immune cells (left panel), immune response pathway (right panel) and other immune-related functions (middle panel). MLTIP scores ordered the scheduling of rows. **B** Alluvial diagram displaying the association between the TCGA cancer type and MLTIP score. **C** Dot plot showing the distributions of MLTIP scores across 30 cancer types, sorted by the median MLTIP score (horizontal line) for each cancer type. **D** Box plots displaying the association of MLTIP score and clinicopathologic features (Stage: stage I, II, III, IV; Gender: female, male; Age: young (<=19), adult (20 ~ 39), pre-aging (40–59), old (>60)) with Wilcoxon test and Kruskal–Wallis test. **E** Forest plot showing the univariate Cox regression analysis of MLTIP score based on OS in TCGA pan-cancer data with two-sided Wald test. The box is presented as HR, and the vertical bar is shown as ± 95% CIs. Green represented the preventive factor, and red represented the risk factor. **F** Kaplan–Meier curves comparing OS between low-risk and low-risk groups predicted by the MLTIP.

We assessed the infiltration of different immune cell subpopulations using the deconvolution methods and enrichment of immune response-related pathways using the ssGSEA across 30 TCGA cancer types, and found that the MLTIP scores highly positively correlated with immune cell infiltration and immune response pathway activation. More specifically, samples with high MLTIP showed increased expression of immune gene signatures (Fig. 5A). This implies that high MLTIP scores could capture active immune tumor microenvironments across various cancer types. In addition, the MLTIP scores vary among patients with the same tumor and between cancer types (Fig. 5B, C), implying general differences in TEX and tumor immunogenicity between different cases of the same tumor and between different tumor types. We further examined the clinical relevance of the MLTIP in pan-cancer, and found that the MLTIP scores decreased significantly for tumors with advanced stages compared with the early stages (Kruskal–Wallis test, *P* < 0.001) (Fig. 5D). There was a significant difference in the MLTIP scores across gender (Wilcoxon test, *P* = 0.024), but no difference for different age groups (Kruskal–Wallis test, *P* = 0.280) (Fig. 5D). The univariate Cox analysis showed that the MLTIP was significantly associated with OS in nine types of cancers (Fig. 5E). However, the prognostic impact of the MLTIP is dissimilar between cancer types. Tumors with high MLTIP phenotype show a significant survival benefit compared with the low MLTIP phenotype in KICH (HR = 0.122, 95% CI, 0.015–0.976, *P* = 0.018), ESCA (HR = 0.545, 95% CI, 0.315–0.941, *P* = 0.027), SKCM (HR = 0.484, 95% CI, 0.352–0.665, *P* < 0.001), BRCA (HR = 0.577, 95% CI, 0.380–0.876, *P* = 0.009), and BLCA (HR = 0.550, 95% CI, 0.312-0.967, *P* = 0.035), while the high MLTIP phenotype is mostly associated with reduced OS in LGG (HR = 1.608, 95% CI, 1.137–2.275, *P* = 0.007), COAD (HR = 2.331, 95% CI, 1.488–3.653, *P* < 0.001), READ (HR = 3.268, 95% CI, 1.465–7.292, *P* = 0.002) and UVM (HR = 6.486, 95% CI, 2.603–16.160, *P* < 0.001) (Fig. 5F).

## Discussion

Immunotherapies have become one of the most promising and critical cancer treatment strategies and have transformed the clinical outcomes for multiple solid tumors [43]. Emerging evidence has highlighted the essential roles of TEX in durable clinical responses to ICB therapy [18, 27]. For example, responders to ICB therapy show amplification of precursor or progenitor exhausted T cells, which is not observed in non-responders [28]. In this study, integrative analysis of ICB-treated bulk and single-cell RNA-seq datasets reveals that transcriptional changes of TEX are associated with the response to ICB immunotherapy. Inspired by these observations, using an ensemble deep-learning computational framework and larger ICB-treated cohorts, we identified 16 essential TEX-related genes (ITGs) associated with ICB efficacy. Some of the 16 ITGs are consistent with previous knowledge that TCF-1, TOX, PD-1, and TIM-3, known key factors for regulating T-cell exhaustion [38, 44–47], are potential predictors for ICB efficacy.

Despite recent efforts and the discovery of potential biomarkers for ICB response, the predictive performance is far from satisfactory. Here, we developed a TEX-dependent predictor of response to ICB immunotherapy (MLTIP) by integrating 16 ITGs via the machine-learning method. The MLTIP was trained from pre-treatment tumor profiles in the Riaz cohort, and displayed high sensitivity and specificity in predicting clinical response to ICB and OS across different cohorts and cancer types. Compared with other state-of-the-art signatures, the MLTIP outperformed them with superior discriminatory power in distinguishing responders from non-responders and achieved a better prognostic performance in different cohorts across multiple cancer types, implying that the MLTIP is robust and may become a competitive tool for identifying patients who benefit from ICB immunotherapy.

Our study has a few limitations. First, we focused primarily on the transcriptional programs of TEX with ICB response due to the unavailability of a multi-omics immunotherapy dataset. Therefore, integrating a multi-omics landscape of intratumoral TEX heterogeneity will improve prediction performance. Second, although TEX is mainly responsible for tumor immune escape and resistance to ICB, it is known that ICB responses were influenced by multi-dimensional interactions between the tumor, the immune system, and other systemic factors [17, 48, 49]; therefore, a combination of other existing biomarkers might be more informative for response prediction. Finally, our study relied on retrospective ICB cohorts limited to several cancer types, future prospective studies covering more cancer types are required to validate our findings.

In conclusion, we developed and validated a TEX-dependent predictor of response to ICB immunotherapy (MLTIP) by integrating 16 ITGs via the machine-learning method. The MLTIP demonstrated superior performance in predicting response to ICB immunotherapy and clinical outcomes before the administration. Therefore, the present study suggests that the TEX-dependent predictor of response to ICB immunotherapy could be a promising tool to guide personalized immunotherapy for cancer patients and improve their clinical outcomes.

## Supplementary information

Supplementary material files

Supplementary Table 1

## Data Availability

The datasets used and/or analyzed during the present study are available from the corresponding author upon reasonable request.
