# Supplementary material for: Deep learning identifies a T-cell exhaustion-dependent transcriptional signature for predicting clinical outcomes and response to immune checkpoint blockade
Source: Oncogenesis. 2023 Jul 11;12(1):37. doi: 10.1038/s41389-023-00482-2 (PMC10336094; doi:10.1038/s41389-023-00482-2)
Supplement: Supplementary file 1 — Supplementary material files [file 41389_2023_482_MOESM1_ESM.docx]

**Supplementary Figures**

**
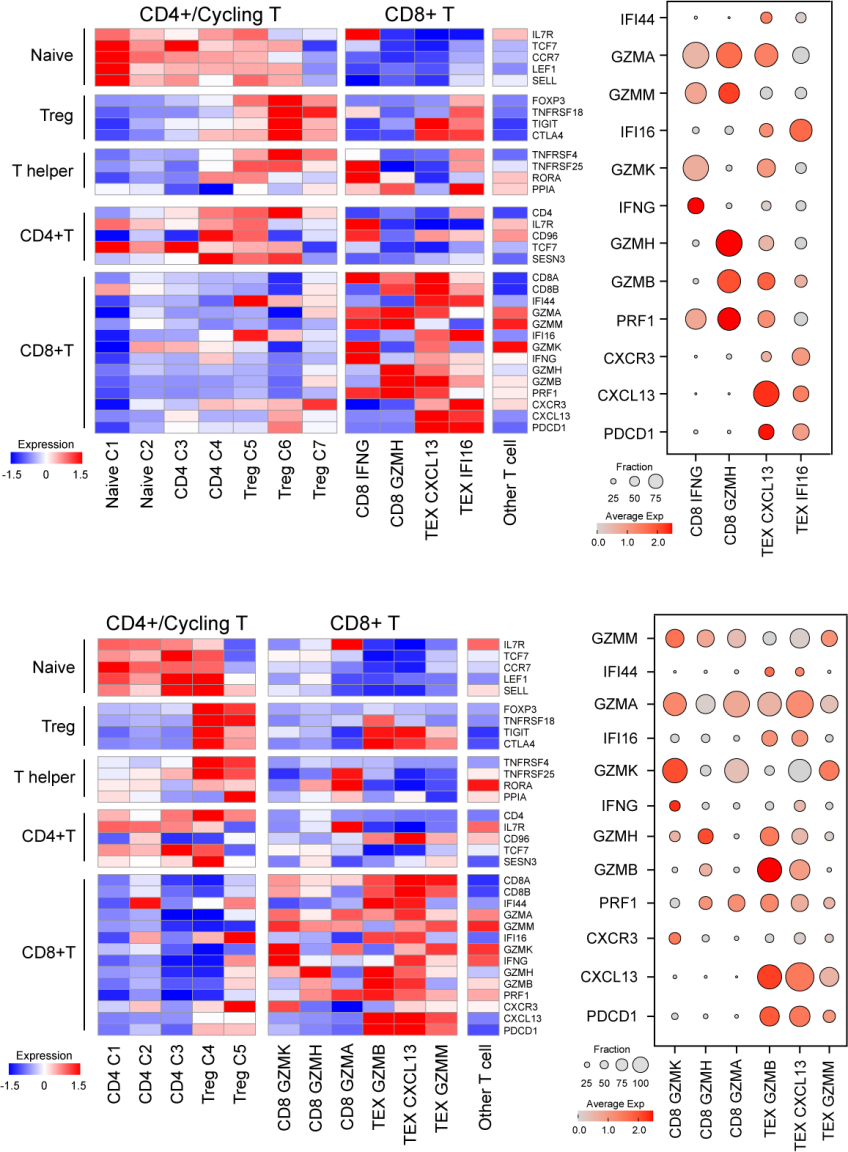
**

**Figure S1.** Heatmaps and dot plots of the z-scored expression pattern of genes highly expressed in corresponding cell clusters in pre-treated and post-treated samples.


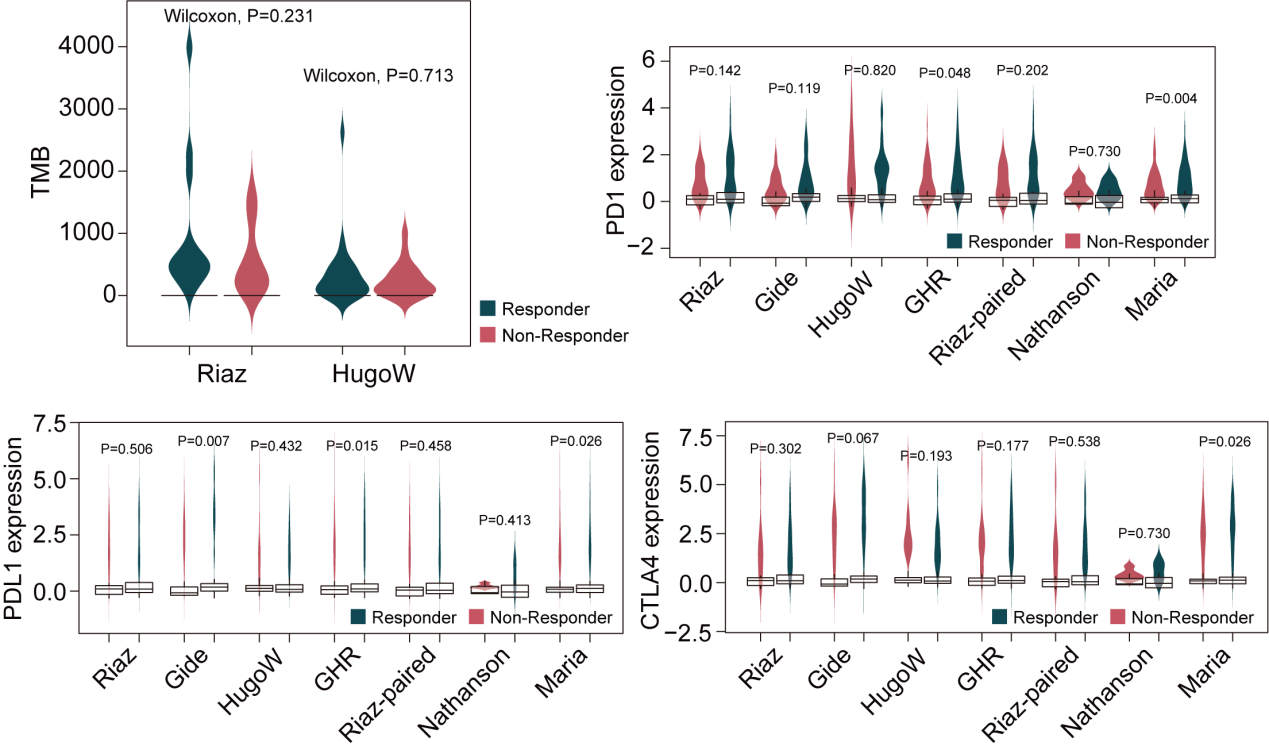


**Figure S2.** Box plots showing the distribution of TMB, PD1, PDL1, and CTLA4 between responders and non-responders.

**Supplementary Tables**

**Supplementary Table 1**. List of 683 genes involved in TEX obtained from previous studies by manual collection.
